# The genome sequence of the holly blue, *Celastrina argiolus* (Linnaeus, 1758)

**DOI:** 10.12688/wellcomeopenres.17478.1

**Published:** 2021-12-14

**Authors:** Alex Hayward, Charlotte Wright, Darwin Tree of Life Barcoding collective, Wellcome Sanger Institute Tree of Life programme, Wellcome Sanger Institute Scientific Operations: DNA Pipelines collective, Tree of Life Core Informatics collective, Darwin Tree of Life Consortium

**Affiliations:** 1 College of Life and Environmental Sciences, Department of Biosciences, University of Exeter, Penryn, UK; 2 Tree of Life, Wellcome Sanger Institute, Cambridge, CB10 1SA, UK

**Keywords:** Celastrina argiolus, holly blue, genome sequence, chromosomal, Lepidoptera

## Abstract

We present a genome assembly from an individual male *Celastrina argiolus*) (the holly blue; Arthropoda; Insecta; Lepidoptera; Lycaenidae). The genome sequence is 499 megabases in span. The majority (99.99%) of the assembly is scaffolded into 26 chromosomal pseudomolecules, with the Z sex chromosome assembled. Gene annotation of this assembly on Ensembl has identified 12,199 protein coding genes.

## Species taxonomy

Eukaryota; Metazoa; Ecdysozoa; Arthropoda; Hexapoda; Insecta; Pterygota; Neoptera; Endopterygota; Lepidoptera; Glossata; Ditrysia; Papilionoidea; Lycaenidae; Polyommatinae; Celastrina; *Celastrina argiolus* (Linnaeus, 1758) (NCBI:txid203782).

## Background

The holly blue, *Celastrina argiolus*, is a widespread butterfly, found throughout the temperate regions of Europe, Asia, North Africa and North America. It is common across the British Isles with the exception of Scotland, where it is absent. Recorded numbers of the butterfly cycles every 4-6 years due to parasitism of the larval form by the larvae of the host-specific ichneumon wasp *Listrodromus nycthemerus* ( Revels, 2006; Revels, 1994). Larvae feed mainly on the flower buds, berries and terminal leaves of holly ( *Ilex aquifolium*) in the spring, and ivy ( *Hedera helix*) in the summer, although they can also use a wide variety of other plants. Adults are distinguished by bright blue wings with pale blue underside and small black spots. In females, the forewings have broad black edges. The species is typically bivoltine and overwinters as pupae. Adults are generalists, feeding on a variety of nectar sources including hawthorn, brambles and Bugle, as well as honey dew. The holly blue has increased in abundance in occurrence over the last fifty years ( Fox *et al.,* 2015) and is considered least threatened in the IUCN Red List (Europe) ( “The IUCN Red List of Threatened Species 2010” 2010). The holly blue has an estimated genome size of 445 Mb based on flow cytometry ( Mackintosh *et al.,* 2019). The karyotype of *C. argiolus* was reported to be 25 by Federley, Lorković, Maeki, and 24 by Bigger, as described in Robinson (1971). ( Bigger, 1961; Federley, 1938; Lorković, 1941; Maeki, 1953; Robinson, 1971). However, the genome assembly described here, confirmed by the presence of telomeric sequence and Hi-C mapping, has a karyotype of 26.

## Genome sequence report

**Figure 1.**
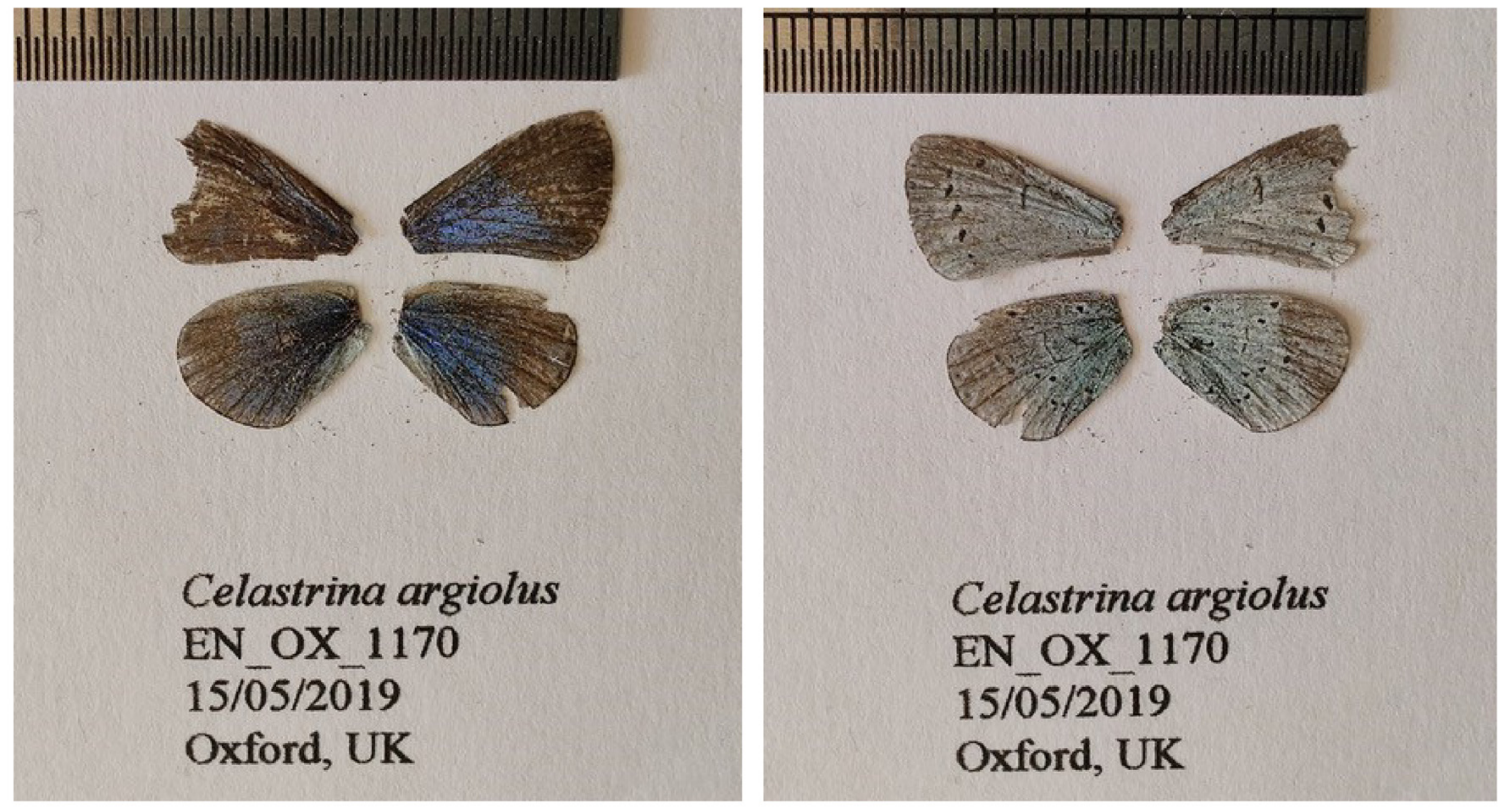
Fore and hind wings of *the Celastrina argiolus* specimen from which the genome was sequenced. Dorsal (left) and ventral (right) surface view of wings from specimen EN_OX_1170 (ilCelArgi3) from Oxford, UK, used to generate Pacific Biosciences and 10X genomics data.

The genome was sequenced from a single male *C. argiolus* ( Figure 1) collected from Oxford, England (latitude 51.74989, longitude 1.22731). A total of 32-fold coverage in Pacific Biosciences single-molecule circular consensus (HiFi) long reads (N50 13 kb) and 69-fold coverage in 10X Genomics read clouds were generated. Primary assembly contigs were scaffolded with chromosome conformation Hi-C data. Manual assembly curation corrected 11 missing/misjoins and removed 3 haplotypic duplications, reducing the assembly length by 0.4%, the scaffold number by 20.6% and the scaffold N50 by 1.3%.

**Figure 2.**
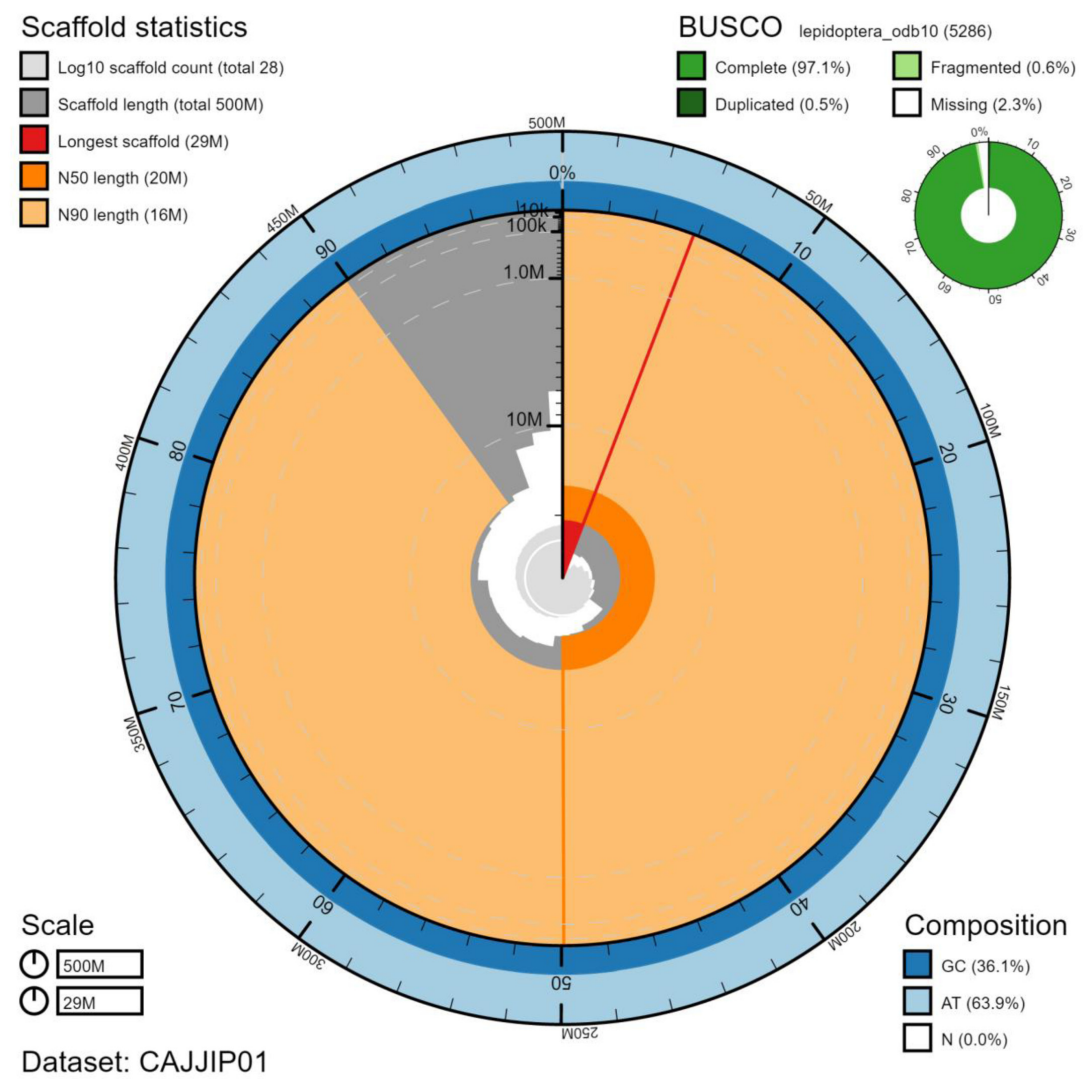
Genome assembly of *Celastrina argiolus*, ilCelArgi3.1: metrics. The BlobToolKit Snailplot shows N50 metrics and BUSCO gene completeness. The main plot is divided into 1,000 size-ordered bins around the circumference with each bin representing 0.1% of the 499,114,119 bp assembly. The distribution of scaffold lengths is shown in dark grey with the plot radius scaled to the longest chromosome present in the assembly (29,052,767 bp, shown in red). Orange and pale-orange arcs show the N50 and N90 chromosome lengths (20,425,925 and 16,318,055 bp), respectively. The pale grey spiral shows the cumulative scaffold count on a log scale with white scale lines showing successive orders of magnitude. The blue and pale-blue area around the outside of the plot shows the distribution of GC, AT and N percentages in the same bins as the inner plot. A summary of complete, fragmented, duplicated and missing BUSCO genes in the lepidoptera_odb10 set is shown in the top right. An interactive version of this figure is available at https://blobtoolkit.genomehubs.org/view/ilCelArgi3.1/dataset/CAJJIP01/snail.

The final assembly has a total length of 499 Mb in 28 sequence scaffolds with a scaffold N50 of 20 Mb ( Table 1). The majority, 99.99%, of assembly sequence was assigned to 26 chromosomal-level scaffolds, representing 25 autosomes (numbered by sequence length), and the Z sex chromosome ( Figure 2– Figure 5; Table 2). The assembly has a BUSCO v5.1.2 ( Simão *et al.,* 2015) completeness of 97.1% using the lepidoptera_odb10 reference set. While not fully phased, the assembly deposited is of one haplotype. Contigs corresponding to the second haplotype have also been deposited.

**Table 1.** Genome data for *Celastrina argiolus*, ilCelArgi3.1.

*Project accession data*	
Assembly identifier	ilCelArgi3.1
Species	*Celastrina agrilous*
Specimen	ilCelArgi3 (genome assembly); ilCelArgi1, ilCelArgi4 (RNA-Seq)
NCBI taxonomy ID	NCBI:txid203782
BioProject	PRJEB41907
BioSample ID	SAMEA7523268
Isolate information	Male, whole organisms
*Raw data accessions*	
PacificBiosciences SEQUEL II	ERR6558180
10X Genomics Illumina	ERR6002602-ERR6002605
Hi-C Illumina	ERR6002606
Illumina polyA RNA-Seq	ERR6002607, ERR6787413
*Genome assembly*	
Assembly accession	GCA_905187575.1
*Accession of alternate haplotype*	GCA_905147145.1
Span (Mb)	499
Number of contigs	137
Contig N50 length (Mb)	8
Number of scaffolds	28
Scaffold N50 length (Mb)	20
Longest scaffold (Mb)	29
BUSCO * genome score	C:97.1%[S:96.7%,D:0.5%],F:0.6%,M:2.3%,n:5286

*BUSCO scores based on the lepidoptera_odb10 BUSCO set using v5.1.2. C= complete [S= single copy, D=duplicated], F=fragmented, M=missing, n=number of orthologues in comparison. A full set of BUSCO scores is available at https://blobtoolkit.genomehubs.org/view/ilCelArgi3.1/dataset/CAJJIP01/busco.

**Figure 3.**
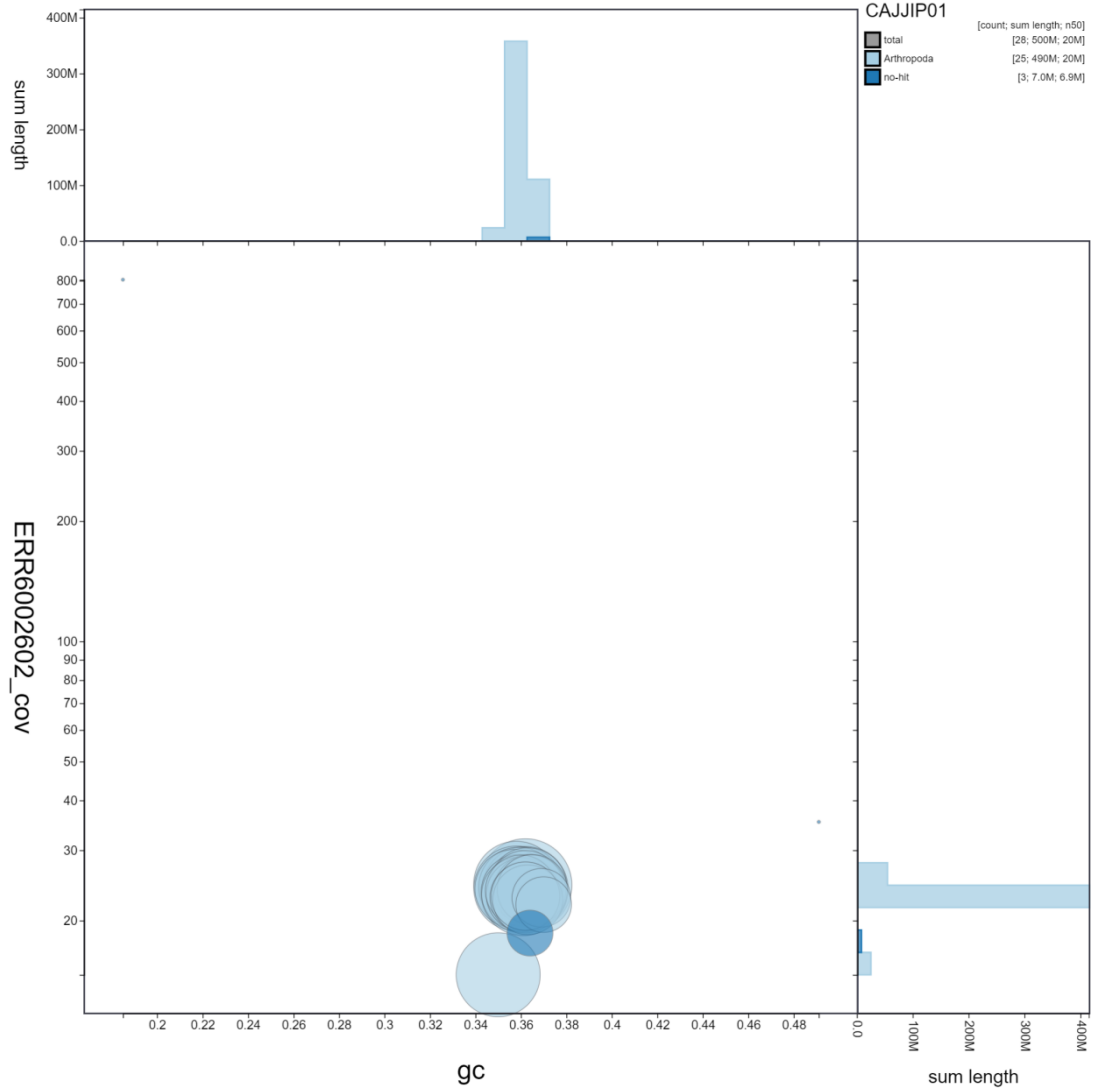
Genome assembly of *Celastrina argiolus*, ilCelArgi3.1: GC coverage. BlobToolKit GC-coverage plot. Scaffolds are coloured by phylum. Circles are sized in proportion to scaffold length. Histograms show the distribution of scaffold length sum along each axis. An interactive version of this figure is available at https://blobtoolkit.genomehubs.org/view/ilCelArgi3.1/dataset/CAJJIP01/blob.

**Figure 4.**
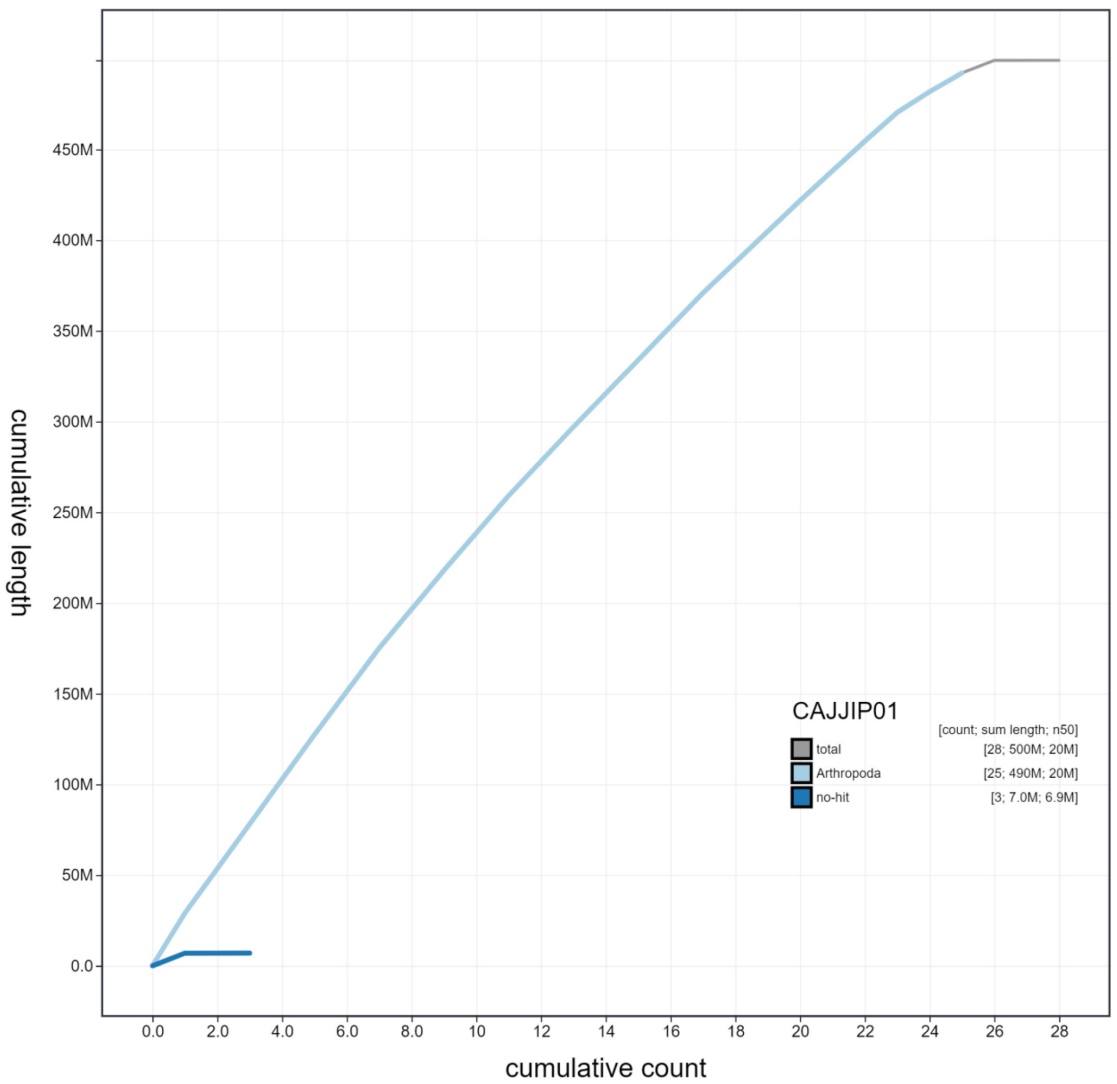
Genome assembly of *Celastrina argiolus*, ilCelArgi3.1: cumulative sequence. BlobToolKit cumulative sequence plot. The grey line shows cumulative length for all scaffolds. Coloured lines show cumulative lengths of scaffolds assigned to each phylum using the buscogenes taxrule. An interactive version of this figure is available at https://blobtoolkit.genomehubs.org/view/ilCelArgi3.1/dataset/CAJJIP01/cumulative.

**Figure 5.**
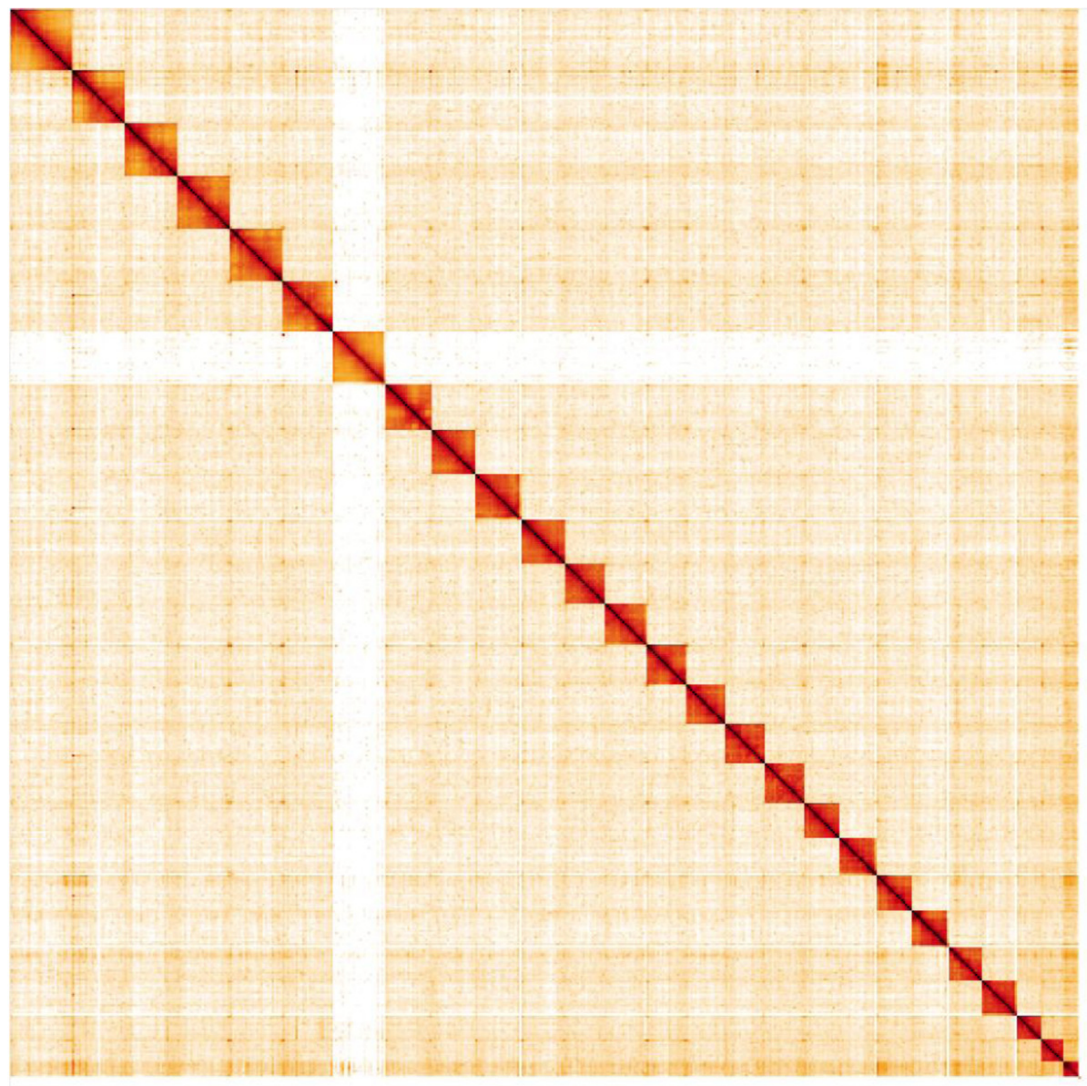
Genome assembly of *Celastrina argiolus*, ilCelArgi3.1: Hi-C contact map. Hi-C contact map of the ilCelArgi3.1 assembly, visualised in HiGlass. Chromosomes are shown in size order from left to right and top to bottom.

**Table 2.** Chromosomal pseudomolecules in the genome assembly of *Celastrina argiolus*, ilCelArgi3.1.

INSDC accession	Chromosome	Size (Mb)	GC%
LR994577.1	1	29.05	36.2
LR994578.1	2	24.85	36
LR994579.1	3	24.55	35.8
LR994580.1	4	24.51	36.2
LR994581.1	5	24.42	35.8
LR994582.1	6	24.04	36.1
LR994584.1	7	21.46	36.1
LR994585.1	8	21.39	35.9
LR994586.1	9	20.68	35.7
LR994587.1	10	20.43	36.3
LR994588.1	11	19.02	36.1
LR994589.1	12	18.92	35.9
LR994590.1	13	18.52	36.4
LR994591.1	14	18.52	36.2
LR994592.1	15	18.42	35.9
LR994593.1	16	18.32	36.1
LR994594.1	17	17.10	36.4
LR994595.1	18	16.97	36
LR994596.1	19	16.88	36.2
LR994597.1	20	16.52	36.5
LR994598.1	21	16.32	36.3
LR994599.1	22	15.74	36.2
LR994600.1	23	11.45	36.9
LR994601.1	24	10.30	37
LR994602.1	25	6.95	36.4
LR994583.1	Z	23.77	35
LR994603.1	MT	18.00	18
-	Unplaced	0.02	49

## Genome annotation report

The ilCelArgi3.1 genome has been annotated using the Ensembl rapid annotation pipeline ( Table 1; GCA_905187575.1). The resulting annotation includes 24,102 transcribed mRNAs from 12,199 protein-coding and 1,981 non-coding genes. There are 1.98 coding transcripts per gene and 8.65 exons per transcript.

## Methods

### Sample acquisition and nucleic acid extraction

Three male *C. argiolus* specimens (ilCelArgi3, genome assembly; ilCelArgi1 and ilCelArgi4, RNA-Seq) were collected from Oxford, England, UK (latitude 51.74989, longitude 1.22731) using a net by Alex Hayward, University of Exeter, who also identified the sample. The samples were frozen at -80°C.

DNA was extracted from the whole organism of ilCelArgi3 at the Wellcome Sanger Institute (WSI) Scientific Operations core from the whole organism using the Qiagen MagAttract HMW DNA kit, according to the manufacturer’s instructions. RNA (from the whole organism of ilCelArgi1 and ilCelArgi4) was extracted in the Tree of Life Laboratory at the WSI using TRIzol, according to the manufacturer’s instructions. RNA was then eluted in 50 μl RNAse-free water and its concentration RNA assessed using a Nanodrop spectrophotometer and Qubit Fluorometer using the Qubit RNA Broad-Range (BR) Assay kit. Analysis of the integrity of the RNA was done using Agilent RNA 6000 Pico Kit and Eukaryotic Total RNA assay.

### Sequencing

Pacific Biosciences HiFi circular consensus and 10X Genomics read cloud DNA sequencing libraries were constructed according to the manufacturers’ instructions. Poly(A) RNA-Seq libraries were constructed using the NEB Ultra II RNA Library Prep kit. DNA and RNA sequencing was performed by the Scientific Operations core at the WSI on Pacific Biosciences SEQUEL II (HiFi), Illumina HiSeq X (10X) and Illumina HiSeq 4000 (RNA-Seq) instruments. Hi-C data were also generated from the whole organism of ilCelArgi3 using the Arima v1.0 kit and sequenced on HiSeq X.

### Genome assembly

Assembly was carried out with Hifiasm ( Cheng *et al.,* 2021); haplotypic duplication was identified and removed with purge_dups ( Guan *et al.,* 2020). One round of polishing was performed by aligning 10X Genomics read data to the assembly with longranger align, calling variants with freebayes ( Garrison & Marth, 2012). The assembly was then scaffolded with Hi-C data ( Rao *et al.,* 2014) using SALSA2 ( Ghurye *et al.,* 2019). The assembly was checked for contamination and corrected using the gEVAL system ( Chow *et al.,* 2016) as described previously ( Howe *et al.,* 2021). Manual curation ( Howe *et al.,* 2021) was performed using gEVAL, HiGlass ( Kerpedjiev *et al.,* 2018) and Pretext. The mitochondrial genome was assembled using MitoHiFi ( Uliano-Silva *et al.,* 2021), which performed annotation using MitoFinder ( Allio *et al.,* 2020). The genome was analysed and BUSCO scores generated within the BlobToolKit environment ( Challis *et al.,* 2020). Table 3 contains a list of all software tool versions used, where appropriate.

**Table 3.** Software tools used.

Software tool	Version	Source
Hifiasm	0.7	Cheng *et al.,* 2021
purge_dups	1.2.3	Guan *et al.,* 2020
SALSA2	2.2	Ghurye *et al.,* 2019
longranger align	2.2.2	https://support.10xgenomics.com/genome-exome/software/pipelines/latest/advanced/other-pipelines
freebayes	1.3.1-17-gaa2ace8	Garrison & Marth, 2012
gEVAL	2016	Chow *et al.,* 2016
HiGlass	1.11.6	Kerpedjiev *et al.,* 2018
PretextView	0.1.x	https://github.com/wtsi-hpag/PretextView
BlobToolKit	2.6.2	Challis *et al.,* 2020

### Gene annotation

The Ensembl gene annotation system ( Aken *et al.,* 2016) was used to generate annotation for the Celastrina argiolus assembly ( GCA_905187575.1; Table 1). The annotation was created primarily through alignment of transcriptomic data to the genome, with gap filling via protein to-genome alignments of a select set of proteins from UniProt ( UniProt Consortium, 2019) and OrthoDB ( Kriventseva *et al.,* 2008). Prediction tools, CPC2 ( Kang *et al.,* 2017) and RNAsamba ( Camargo *et al.,* 2020), were used to aid determination of protein coding genes.

## Data availability

European Nucleotide Archive: Celastrina argiolus (holly blue) genome assembly, ilCelArgi3. Accession number PRJEB41907: https://www.ebi.ac.uk/ena/browser/view/PRJEB41907

The genome sequence is released openly for reuse. The *C. argiolus* genome sequencing initiative is part of the Darwin Tree of Life (DToL) project. All raw sequence data and the assembly have been deposited in INSDC databases. Raw data and assembly accession identifiers are reported in Table 1.

## Author information

Members of the Darwin Tree of Life Barcoding collective are listed here: https://doi.org/10.5281/zenodo.4893704.

Members of the Wellcome Sanger Institute Tree of Life programme collective are listed here: https://doi.org/10.5281/zenodo.4783586.

Members of Wellcome Sanger Institute Scientific Operations: DNA Pipelines collective are listed here: https://doi.org/10.5281/zenodo.4790456.

Members of the Tree of Life Core Informatics collective are listed here: https://doi.org/10.5281/zenodo.5013542.

Members of the Darwin Tree of Life Consortium are listed here: https://doi.org/10.5281/zenodo.4783559.

## References

[ref-1] Aken BL Ayling S Barrell D : The Ensembl Gene Annotation System. *Database (Oxford).* 2016;2016:baw093. 10.1093/database/baw093 27337980 PMC4919035

[ref-2] Allio R Schomaker-Bastos A Romiguier J : MitoFinder: Efficient Automated Large-Scale Extraction of Mitogenomic Data in Target Enrichment Phylogenomics. *Mol Ecol Resour.* 2020;20(4):892–905. 10.1111/1755-0998.13160 32243090 PMC7497042

[ref-3] Bigger T : Chromosome Numbers of Lepidoptera. Part II. *Entomologist’s Gazette.* 1961;12:85–89.

[ref-4] Camargo AP Sourkov V Pereira GAG : RNAsamba: Neural Network-Based Assessment of the Protein-Coding Potential of RNA Sequences. *NAR Genom Bioinform.* 2020;2(1):lqz024. 10.1093/nargab/lqz024 33575571 PMC7671399

[ref-5] Challis R Richards E Rajan J : BlobToolKit - Interactive Quality Assessment of Genome Assemblies. *G3 (Bethesda).* 2020;10(4):1361–74. 10.1534/g3.119.400908 32071071 PMC7144090

[ref-6] Cheng H Concepcion GT Feng X : Haplotype-Resolved *de Novo* Assembly Using Phased Assembly Graphs with Hifiasm. *Nat Methods.* 2021;18(2):170–75. 10.1038/s41592-020-01056-5 33526886 PMC7961889

[ref-7] Chow W Brugger K Caccamo M : gEVAL - a Web-Based Browser for Evaluating Genome Assemblies. *Bioinformatics.* 2016;32(16):2508–10. 10.1093/bioinformatics/btw159 27153597 PMC4978925

[ref-8] Federley H : Chromosomenzahlen Finnlän-Discher Lepidopteren. *Hereditas.* 1938;24(4):397–464. 10.1111/j.1601-5223.1938.tb03219.x

[ref-9] Fox R Brereton TM Asher J : The State of the UK’s Butterflies 2015.2015. Reference Source

[ref-10] Garrison E Marth G : Haplotype-Based Variant Detection from Short-Read Sequencing.arXiv: 1207.3907.2012. Reference Source

[ref-11] Ghurye J Rhie A Walenz BP : Integrating Hi-C Links with Assembly Graphs for Chromosome-Scale Assembly. *PLoS Comput Biol.* 2019;15(8):e1007273. 10.1371/journal.pcbi.1007273 31433799 PMC6719893

[ref-12] Guan D McCarthy SA Wood J : Identifying and Removing Haplotypic Duplication in Primary Genome Assemblies. *Bioinformatics.* 2020;36(9):2896–98. 10.1093/bioinformatics/btaa025 31971576 PMC7203741

[ref-13] Howe K Chow W Collins J : Significantly Improving the Quality of Genome Assemblies through Curation. *GigaScience.* 2021;10(1):giaa153. 10.1093/gigascience/giaa153 33420778 PMC7794651

[ref-14] Kang YJ Yang DC Kong L : CPC2: A Fast and Accurate Coding Potential Calculator Based on Sequence Intrinsic Features. *Nucleic Acids Res.* 2017;45(W1):W12–16. 10.1093/nar/gkx428 28521017 PMC5793834

[ref-15] Kerpedjiev P Abdennur N Lekschas F : HiGlass: Web-Based Visual Exploration and Analysis of Genome Interaction Maps. *Genome Biol.* 2018;19(1):125. 10.1186/s13059-018-1486-1 30143029 PMC6109259

[ref-16] Kriventseva EV Rahman N Espinosa O : OrthoDB: The Hierarchical Catalog of Eukaryotic Orthologs. *Nucleic Acids Res.* 2008;36(Database issue):D271–75. 10.1093/nar/gkm845 17947323 PMC2238902

[ref-17] Lorković Z : Die Chromosomezahlen in Der Spermatogenese Der Tagfalter. *Chromosoma.* 1941;2:155–91.

[ref-18] Mackintosh A Laetsch DR Hayward A : The Determinants of Genetic Diversity in Butterflies. *Nat Commun.* 2019;10(1):3466. 10.1038/s41467-019-11308-4 31371715 PMC6672018

[ref-19] Maeki K : Chromosome Numbers of Some Butterflies. *Jap J Genet.* 1953;28(1):6–7. Reference Source

[ref-20] Rao SSP Huntley MH Durand NC : A 3D Map of the Human Genome at Kilobase Resolution Reveals Principles of Chromatin Looping. *Cell.* 2014;159(7):1665–80. 10.1016/j.cell.2014.11.021 25497547 PMC5635824

[ref-21] Revels R : The Rise and Fall of the Holly Blue Butterfly. *British Wildlife.* 1994;5:236. Reference Source

[ref-22] Revels R : More on the Rise and Fall of the Holly Blue. *British Wildlife.* 2006;17(6):419. Reference Source

[ref-23] Robinson R : Lepidoptera Genetics.Oxford: Pergamon Press,1971. Reference Source

[ref-24] Simão FA Waterhouse RM Ioannidis P : BUSCO: Assessing Genome Assembly and Annotation Completeness with Single-Copy Orthologs. *Bioinformatics.* 2015;31(19):3210–12. 10.1093/bioinformatics/btv351 26059717

[ref-25] The IUCN Red List of Threatened Species 2010.e.T174285A7043226.2010. Reference Source

[ref-26] Uliano-Silva M Nunes JGF Krasheninnikova K : marcelauliano/MitoHiFi: mitohifi_v2.0.2021. 10.5281/zenodo.5205678

[ref-27] UniProt Consortium: UniProt: A Worldwide Hub of Protein Knowledge. *Nucleic Acids Res.* 2019;47(D1):D506–15. 10.1093/nar/gky1049 30395287 PMC6323992

