# Supplementary material for: The genome sequence of the holly blue, Celastrina argiolus (Linnaeus, 1758)
Source: Wellcome Open Res. 2021 Dec 14;6:340. [Version 1] doi: 10.12688/wellcomeopenres.17478.1 (PMC8729184; doi:10.12688/wellcomeopenres.17478.1)
Supplement: Peer review history

# Peer review: Reviewer response for version 1

**DOI:** 10.21956/wellcomeopenres.19326.r47621

**Published:** 2022-01-04

**Authors:** Bin Liang

**Affiliations:** 1 College of Life Sciences, Inner Mongolia University, Hohhot, China

The authors submitted a chromosome-level genome assembly of the holly blue, *Celastrina argiolus*. In the method part, the authors scientifically described how they finished sampling, genome DNA and RNA sequencing, assembly and annotation. In this good work, the authors combined several popular sequencing techniques to guarantee the assembly quality, including a long-read sequencing, Pacbio HiFi, 10X Genomics sequencing, RNA-seq and Hi-C mapping. All results are perfect. In order to verify the identity of the sample, I suggest author had better conduct nucleotide blast in ncbi database using their assembly of the mitochondrial genome. I believe they already did it and can show sequence identity comparing with published mitochondrial DNA of *Celastrina argiolus*. All protocols and software are described clearly to ensure the reader can follow the authors' performance.

Are sufficient details of methods and materials provided to allow replication by others?

Yes

Is the rationale for creating the dataset(s) clearly described?

Yes

Are the datasets clearly presented in a useable and accessible format?

Yes

Are the protocols appropriate and is the work technically sound?

Yes

Reviewer Expertise:

Phylogeny, Comparative genomics, Mitochondrial genome

I confirm that I have read this submission and believe that I have an appropriate level of expertise to confirm that it is of an acceptable scientific standard.

# Peer review: Reviewer response for version 1

**DOI:** 10.21956/wellcomeopenres.19326.r47619

**Published:** 2021-12-16

**Authors:** Carles Lalueza-Fox

**Affiliations:** 1 Institute of Evolutionary Biology (CSIC-UPF), Barcelona, Spain

The paper presents the assembly and annotation of a common Lycaenidae butterfly, the holly blue (Celastrina argiolus). The methods are clearly written and well described, and the standard parameters of quality (such as N50 of 20Mb or BUSCO over 97%) indicates this a sound annotated genome that can be of utility not only to explore the holly blue's own evolutionary traits, but also, by means of its widespread distribution, to explore adaptive strategies to different environmental conditions. The authors cite the relevant literature for this common and yet understudied butterfly; precisely in Mackintosh et al. (2019) ^1^, the authors compare the genetic diversity across a large dataset of butterflies, finding that the holly blue, despite being a generalist, has a relatively low genetic diversity (as compared to other butterflies, some of them more geographically restricted). I think this could also be mentioned in the Introduction because, obviously, a reference genome for a species with low diversity is scientifically much more valuable than one for a species that can be highly structured and displays high diversity parameters.

Are sufficient details of methods and materials provided to allow replication by others?

Yes

Is the rationale for creating the dataset(s) clearly described?

Yes

Are the datasets clearly presented in a useable and accessible format?

Yes

Are the protocols appropriate and is the work technically sound?

Yes

Reviewer Expertise:

Evolutionary genomics

I confirm that I have read this submission and believe that I have an appropriate level of expertise to confirm that it is of an acceptable scientific standard.

## References

[1] : The determinants of genetic diversity in butterflies. *Nature Communications* .2019;10(1) : 10.1038/s41467-019-11308-4 10.1038/s41467-019-11308-4 PMC667201831371715
